# Meeting in Manchester

**Published:** 1918-05-04

**Authors:** 


### Meeting in Manchester.

A very successful meeting was held at the Manchester Royal Infirmary, in the Out-patients' Hall, at 4.30 p.m., on Friday, April 26. Much interest has been aroused in Manchester both in the College of Nursing and the Nation's Fund for Nurses. There was a very representative gathering, and a good muster of members of both the medical and nursing professions. The large hall was nearly full.

**Sir WILLIAM COBBETT,** as Chairman of the Board of Management, welcomed the speakers, Sir Arthur Stanley and Dame May Whitty, to the meeting. He alluded to the splendid work done by nurses during the war, and advised all nurses to join the College and to influence others to do the same and to give it their whole-hearted support. He was firmly convinced that it was the right thing to do. He then went on to explain that the object of the meeting was to raise funds to support the endowment of the College and to promote a Benevolent Fund for nurses.

**Sir ARTHUR STANLEY** spoke of the pleasure it gave him to come to Manchester again in connection with the College of Nursing, and stated that the membership of the College was increasing rapidly. He endorsed Sir William Cobbett's remarks, and urged all nurses who were already members to use their influence to get other nurses to join. Reference was made to the divided parties for State Registration, but Sir Arthur Stanley spoke very hopefully as to there being an agreed Bill in the not far away future. He congratulated Manchester on its College Centre and its activity, and spoke of the great help these Centres would be. That at Manchester, in particular, had made such an excellent start that it ought to have a very good future before it. He went on to explain the College of Nursing and the Nation's Fund for Nurses, paying a glowing tribute to the work which had already been done by the British Women's Hospital Committee for the Star and Garter Hospital and for disabled soldiers, and how fortunate he considered the College was to have their assistance.

**Dame MAY WHITTY,** as Chairman of the British Women's Hospital Committee, gave a detailed account of the work of the committee and their interest in matters concerning the women of the country, but particularly nurses. In describing the plans they adopted in working for the Nation's Fund for Nurses, she spoke very feelingly on the question as to whether appealing for help for the Nation's Fund was asking the nurses to receive charity. It *was* charity, but only in its broad meaning of "Love." The nation was filled with gratitude and love for what the nurses had done and were still doing in this great war. Dame May Whitty was sure that the money would be forthcoming both to help, in the first place, the endowment of the College and its buildings and the higher education of nurses, and, secondly, for the Benevolent Fund for nurses who required assistance.

**Sir WILLIAM MILLIGAN,** in supporting the College of Nursing very strongly, and also the Nation's Fund for Nurses, proposed a resolution that a committee should be formed to carry out the organising of this Fund in Manchester. Mr. Percy Musgrave, Chairman of Bolton Infirmary, seconded the resolution.

**Sir WILLIAM COBBETT** put the resolution to the meeting, and it was carried unanimously, a list of names of those willing to serve on the committee being forthcoming at once.

**Sir DANIEL McCABE** proposed a hearty vote of thanks to the speakers, which was seconded by Miss Fletcher, R.R.C., matron of Whitworth Street Military Hospital, Manchester.

### MEETING AT TRURO.

#### A Local Centre Urged.

A meeting was held in the Town Hall, Truro, on April 29. The Mayor presided over a representative audience, matrons and nurses from all parts of the county being present. Miss Alison Garland explained the aims of the College and the Nation's Fund for Nurses, and a substantial collection for this Fund followed her rousing appeal. Mr. D. H. Shilson, President of the Royal Cornwall Infirmary, and Dr. Panting urged the claims of the College and the Nation's Fund. Harriet, Lady Trelawney, in proposing a vote of thanks to the speakers, gave interesting reminiscences of the old days before the aid of nurses was available in Cornwall. Mrs. Polwhele seconded the resolution. Miss Bessie Chaff, Matron of the Royal Cornwall Infirmary, proposed a vote of thanks to the Mayor, and urged the desirability of forming a local centre of the College in Cornwall. Miss Riden, Superintendent of the Cornwall County Nursing Association, seconded the vote and heartily endorsed Miss Chaff's suggestion.

**☞ Owing to pressure on our space the report of the Leicester Meeting on behalf of the National Tribute Fund is unavoidably held over.**

